# The Dorsolateral Periaqueductal Gray and Its Role in Mediating Fear Learning to Life Threatening Events

**DOI:** 10.1371/journal.pone.0050361

**Published:** 2012-11-28

**Authors:** Grasielle C. Kincheski, Sandra R. Mota-Ortiz, Eloisa Pavesi, Newton S. Canteras, Antônio P. Carobrez

**Affiliations:** 1 Departamento de Farmacologia, CCB, Universidade Federal de Santa Catarina, Florianópolis, Santa Catarina, Brazil; 2 Laboratório Bases Neurais do Comportamento, Universidade Cidade de São Paulo, São Paulo, Brazil; 3 Departamento de Anatomia, Instituto de Ciências Biomédicas, Universidade de São Paulo, São Paulo, Brazil; Tokai University, Japan

## Abstract

The dorsolateral column of the periaqueductal gray (dlPAG) integrates aversive emotional experiences and represents an important site responding to life threatening situations, such as hypoxia, cardiac pain and predator threats. Previous studies have shown that the dorsal PAG also supports fear learning; and we have currently explored how the dlPAG influences associative learning. We have first shown that N-methyl-D-aspartate (NMDA) 100 pmol injection in the dlPAG works as a valuable unconditioned stimulus (US) for the acquisition of olfactory fear conditioning (OFC) using amyl acetate odor as conditioned stimulus (CS). Next, we revisited the ascending projections of the dlPAG to the thalamus and hypothalamus to reveal potential paths that could mediate associative learning during OFC. Accordingly, the most important ascending target of the dlPAG is the hypothalamic defensive circuit, and we were able to show that pharmacological inactivation using β-adrenoceptor blockade of the dorsal premammillary nucleus, the main exit way for the hypothalamic defensive circuit to thalamo-cortical circuits involved in fear learning, impaired the acquisition of the OFC promoted by NMDA stimulation of the dlPAG. Moreover, our tracing study revealed multiple parallel paths from the dlPAG to several thalamic targets linked to cortical-hippocampal-amygdalar circuits involved in fear learning. Overall, the results point to a major role of the dlPAG in the mediation of aversive associative learning via ascending projections to the medial hypothalamic defensive circuit, and perhaps, to other thalamic targets, as well. These results provide interesting perspectives to understand how life threatening events impact on fear learning, and should be useful to understand pathological fear memory encoding in anxiety disorders.

## Introduction

In humans, activation of the periaqueductal gray (PAG) has been correlated with fear and anger manifestations in normal volunteers [1] and distress episodes in post traumatic stress disorder (PTSD) patients submitted to cue reminders of the trauma [2]. Additionally, recent structural neuroimaging data in human patients suggested the involvement of the PAG in panic disorders [3], [4]. In line with this view, there is a growing body of evidence suggesting the PAG as a key locus to integrate panic-like responses. In neurosurgical procedure in humans, stimulation of the dorsal PAG has been shown to elicit feelings of fear, impending death and apprehensive avoidance [5], [6], [7]. Across different species, stimulation of the dorsal PAG is known to induce panic-like responses [8], [9], [10] and is thought to work as a reliable animal model of panic attacks [11], [12], [13]. In rodents, the dorsolateral PAG (dlPAG) is particularly responsive to life threatening events, such as predator cues [14], [15], interoceptive signals of hypoxia [16], [17] and cardiac pain [18]. Conversely, dlPAG stimulation evokes full blown defense, including freezing and flight behavior, as well as the accompanying sympathetic responses, such as exophtalmos, and increased heart rate and blood pressure [9], [10].

Apart from responding to life threatening events and organizing fear responses, the dorsal PAG has been shown to support fear learning. Dorsal PAG stimulation has been used as a US in contextual conditioning paradigms [19], [20], and it has been shown that the integrity of the dlPAG glutamatergic circuit is necessary to support fear conditioning using chemical stimulation of the dorsal premammillary nucleus as a US to mimic predator exposure [21]. Altogether, the dlPAG emerges as a key site to respond to life threatening events and, at the same time, to influence fear learning.

One of the most common animal models of PTSD involves exposing a rodent to a predator threat, a potentially life-threatening situation that provides PTSD-like behavioral responses, including resistance of the traumatic memories to extinction, hyperarousal and social withdrawal [22], [23]. The investigation on the putative mechanisms underlying the dlPAG participation in fear conditioning seems an interesting approach to explore how life threatening events impact on fear learning, and should be particularly useful to understand pathological fear memories encoding in patients suffering from PTSD. In PTSD patients, olfactory cues associated to the traumatic event can be engraved as emotional memory able to precipitate vivid revival of the trauma, resulting in higher order conditioning to places and contexts [24], [25], [26]. Considering the importance of olfactory stimuli to mark traumatic events, we have used olfactory fear conditioning (OFC) to investigate how the dlPAG influences fear learning.

The OFC has been obtained by pairing fear-like state induced by N-methyl-D-aspartate (NMDA) dlPAG stimulation as unconditioned stimulus (US) with a neutral odor as conditioned stimulus (CS). Neutral olfactory cues have been employed as CS in fear conditioning paradigms [27], [28], [29], [30]. Rodents, in particular, have specific olfactory anatomical adaptations enabling discrimination among odorants, a fact that certainly contributes to enhance learning and memory capacities [31], [32].

In the present study, we have first investigated whether fear-like state induced by N-methyl-D-aspartate (NMDA) dlPAG stimulation would be a valuable US in an OFC paradigm. Next, we revisited the ascending projections of the dlPAG to the thalamus and hypothalamus to reveal potential paths that could mediate associative learning during OFC. And, finally, we tested how pharmacological inactivation of the main dlPAG ascending target, the medial hypothalamic defensive circuit, would interfere in the OFC acquisition. Overall, the results point to a major role of the dlPAG in the mediation of aversive associative learning process via ascending projections to medial hypothalamic targets, and perhaps, to other thalamic targets, as well.

**Figure 1:**
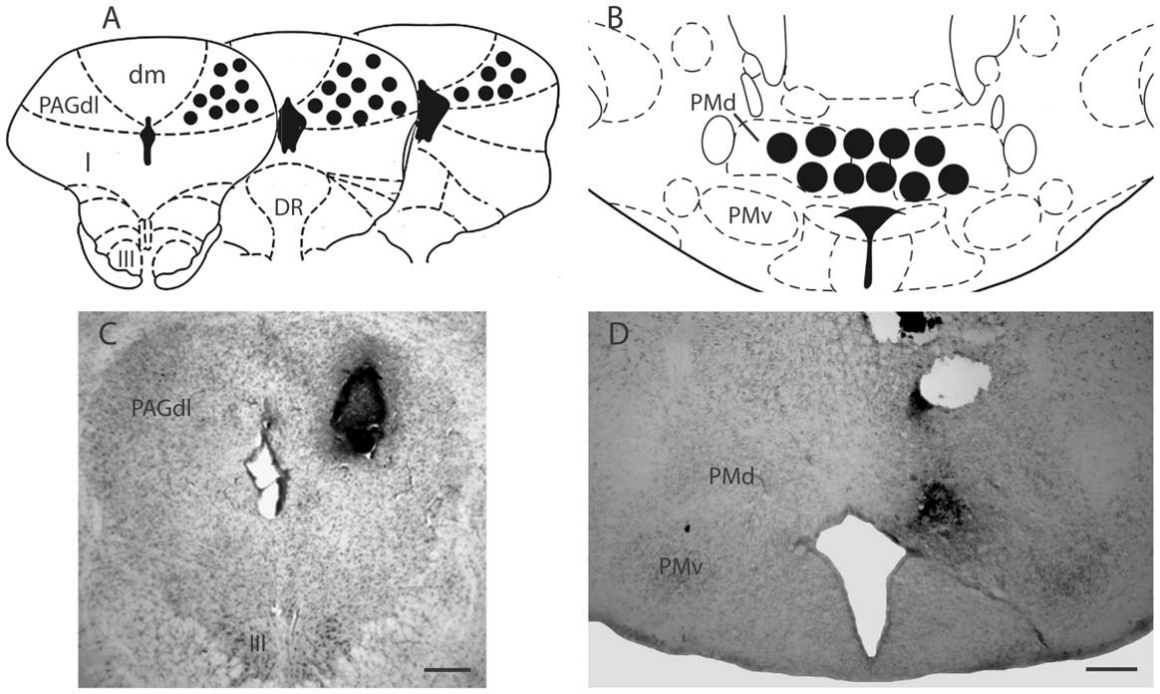
Histological analysis of the injection site. A, B – Schematic plotting onto a standard drawing of *The rat brain in stereotaxic coordinates* [33] showing the approximate location of the injection cannula tips centered in the dlPAG (A) and the PMd (B). Due to the large number of overlaps, the number of plotted points is lower than the number of subjects actually injected in these regions. C – Bright-field photomicrograph showing Evans blue-stained injection cannula placement in a representative animal that received NMDA 100 pmol into the dlPAG. D – Bright-field photomicrograph showing Evans blue-stained injection cannula placement in a representative animal that received ATE 40 nmol into the PMd region. Abbreviations: DR – dorsal nucleus raphé; III – oculomotor nucleus; PAGdl, dm, l – periaqueductal gray, dorsolateral, dorsomedial, and lateral parts; PMd – dorsal premammillary nucleus; PMv – ventral premammillary nucleus. Scale bars = 250 µm.

## Materials and Methods

### Ethics Statement

All the experiments were conducted according to the Society for Neuroscience ethical guidelines for care and use of laboratory animals and were approved by the Ethic Committee on The Use of Laboratory Animals (CEUA) from *Universidade Federal de Santa Catarina* (Protocol number 23080.0055752/2006-64). All surgery was performed under xylazine plus ketamine anesthesia, and all efforts were made to minimize suffering.

### Animals and Housing

Male Wistar rats (n = 116) weighing 300–50 g at the beginning of each experiment were housed in groups of three per cage (50×30×15 cm) in a temperature-controlled room (23±1°C) under standard laboratory conditions with free access to food and water and a 12 h light/12 h dark cycle (lights on at 7∶00 a.m.).

### Stereotaxic Surgery and dlPAG Infusion Procedure

Ten days before the beginning of the experiments, all subjects used in the behavioral tests were submitted to a stereotaxic surgery for cannula implant into the dlPAG. The rats were anaesthetized (i.p.) with 1.5 ml/kg of a mixture (v/v) containing xylazine (10 mg/ml; Dopaser®, São Paulo, Brazil) plus ketamine (58 mg/ml; Dopalen®, São Paulo, Brazil) and fixed in a stereotaxic frame (Stoelting Co., U.S.A). Xylocaine (0.1 ml, 2 mg/ml; Probem, Brazil) was subcutaneously injected into the scalp and a longitudinal incision was made. A stainless steel guide cannula (0.7 mm external diameter; 13 mm length) was implanted unilaterally aimed at the dlPAG (coordinates from bregma: ML = 1.9 mm; AP = −7.6 mm; DV = −2.0 mm from the skull surface at an angle of 22°), according to *The rat brain in stereotaxic coordinates* [33]. The cannula was attached to the bone with stainless steel screws and acrylic cement. A stylet inside the guide cannula prevented obstruction. At the end of the surgery, the subjects received an intramuscular injection of Pentabiotic (60,000 IU, 0.2 ml; Fort Dodge, Brazil) and a subcutaneous injection (10 mg/ml) of the anti-inflammatory and analgesic Banamine (flunixinmeglumine, 2.5 mg/kg; Schering-Plough, Brazil).

N-Methyl-D-aspartic acid (NMDA; Sigma, St. Louis, USA) was dissolved in 0.1 M phosphate-buffered saline (PBS; pH 7.4), which alone served as vehicle control. The doses of NMDA (25, 50 and 100 pmol), the volume (0.2 µl) and rate (0.6 µl/min) of infusion were chosen based on previous studies [10], [34], [35].

For intracerebral drug administration, subjects were gently held, the stylet was removed and a stainless steel needle (16.2 mm long with 0.35 mm external diameter) was inserted into the guide cannula. The needle was connected to a 5-µl Hamilton microsyringe by a polyethylene tubing (PE10; Clay Adams, USA) and the injections were performed using an automated infusion pump (Insight Ltda, Ribeirão Preto, Brazil). The forward movement of a small air bubble inside the polyethylene tubing was taken as evidence of drug flow.

### Apparatuses and Behavioral Measures

Experimental procedure comprised five sessions, spaced 24 h apart, in two different apparatuses, i.e., a conditioning chamber and an odor box. The conditioning chamber (50×26×35 cm) was constructed with stainless steel walls and a grid floor composed of 1-cm spaced stainless steel bars. The odor box (60×26×40 cm) was made up of black Plexiglas and consisted of an open compartment (40×26×40 cm) and an enclosed (roofed) compartment (20×26×40 cm). A 6×6 cm hole allowed the rats to move through both compartments. The frontal side of the chamber was made of clear Plexiglas allowing a video camera and corresponding DVD system to record the subject’s behavior. All sessions were performed in a sound-attenuated room with illumination level of 4–11 lux; and all the experiments were performed during the diurnal phase, between 13∶00 and 17∶00 h. The olfactory stimulus used was 250 µl of 5% amyl acetate (AMYL 99+% SAFC Supply Solutions; Sigma, St. Louis, USA) diluted in propylene glycol. The choice of amyl acetate odor at a 5% dilution as a CS was based on previous studies [30], [36]. After each session, and between subjects, the apparatuses were cleaned with a 10% alcohol-water solution.

On day 1, each rat was placed in the conditioning chamber and allowed to freely explore it for 5 min. On the following day, immediately after receiving a dlPAG microinjection, subjects were placed in the conditioning chamber saturated with AMYL. For AMYL saturation, a filter paper containing 250 µl of 5% AMYL solution was placed in the compartment under the grid floor. The time of the conditioning session varied (5 or 10 min). During this phase, the time spent in freezing behavior and the episodes of flight and jumping, as the result of dPAG-NMDA infusion, were scored.

In order to assure the selectivity of the AMYL odor as the CS, the expression of OFC was evaluated in the odor box (three sessions of 10 min each). In the first session, each rat was placed in the odor box without the CS, to habituate to the apparatus, and the behavioral scoring represented the baseline level during the familiarization session. On the following day, the subjects were replaced in the odor box now containing the filter paper with AMYL in a perforated acrylic box (6×9×1 cm), displayed at the far end of the open compartment (first-order conditioning, CS1). Twenty-four hours later, rats returned to the odor box without the odor source, and were tested for second-order conditioning (CS2) as a result of AMYL odor (CS1) and contextual pairing. During both the familiarization and the CS2 session, a clean odorless filter paper inserted into the acrylic box was used only as a visual mark.

Behavioral defensive reactions, observed during the odor box exposure, were defined based on previous data from several laboratories that showed the same profile of responses in rats submitted to cat odor exposure [37], [38] as well as in a previous OFC study [21]. Therefore, the same fear-related behavioral responses were measured during exposure to the odor box which include: a) approach time - the amount of time the rats spent near (within 7 cm) the odor source; b) hide time - the amount of time spent in the enclosed compartment; and c) head-out time - the amount of time spent stretching out from the enclosed compartment toward the open compartment.

**Table 1:** Percentage of rats exhibiting Flight or Jumping during the first min following PBS or NMDA infusion into dlPAG.

	% Subjects		
Treatments	N	Flight	Jumping
PBS	8	0	0
NMDA 25	8	25.0	0
NMDA 50	8	37.5	12.5
NMDA 100	8	62.5	25.0

**Figure 2:**
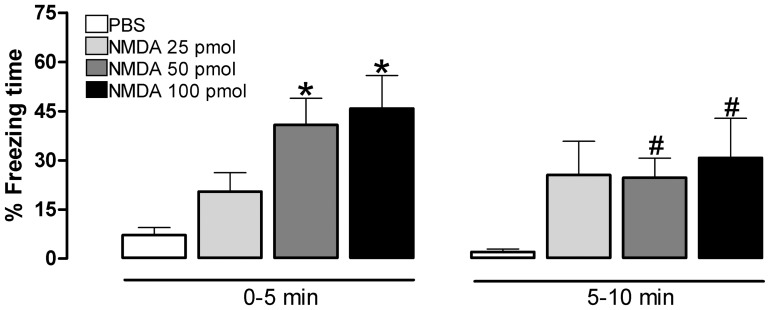
Freezing response during conditioning session. Freezing responses of rats following PBS (N = 8) or NMDA [25 (N = 8), 50 (N = 8) or 100 (N = 8) pmol] infusion in the dlPAG and placed in a conditioning box with an olfactory CS. Plotted values represent mean (+SEM) collapsed in two subsequent 5-min periods. *p<0.05, compared to PBS, during the first 5-min period. #p<0.05 compared to same group during the first 5-min period (repeated measures ANOVA followed by Newman-Keuls test).

#### Experiment 1

Experiment 1 was conducted to investigate if NMDA stimulation of the dlPAG would work as a useful US capable of supporting OFC, and to test the optimal NMDA dose to produce OFC. Twenty-four hours after a familiarization session (5 min) in the conditioning chamber, the subjects were microinjected into the dlPAG with PBS (n = 8) or crescent doses of NMDA (25 pmol, n = 8; 50 pmol, n = 8; or 100 pmol, n = 8) and immediately exposed to the conditioning chamber with amyl acetate odor, for 10 minutes. During the conditioning session, the amount of time the rats spent freezing, as well as the occurrences of flight and jumping, was scored during a ten min period. Twenty four hours later, the defensive behavior expression was measured in the odor box, in three subsequent sessions, 24 h apart (familiarization, CS1 and CS2), as described above.

#### Experiment 2

In order to test for the selectivity of the dlPAG NMDA injection in supporting OFC, subjects receiving dlPAG injections of NMDA 100 pmol (NMDA/odor, n = 10) or PBS (PBS/odor, n = 8), paired with AMYL odor, during the conditioning session, were compared with two other groups microinjected into the dlPAG with PBS (PBS/no odor, n = 8) or NMDA 100 pmol (NMDA/no odor, n = 8), not paired with odor in the conditioning box. An additional group receiving injections of NMDA 100 pmol outside the dlPAG (in the adjacent midbrain reticular nucleus, NMDA-out/odor, n = 8) was also paired with AMYL odor during the conditioning session. Twenty four hours later, the defensive behavior expression was measured in three subsequent sessions (familiarization, CS1 and CS2, 24 h apart) in the odor box, as described above.

**Figure 3:**
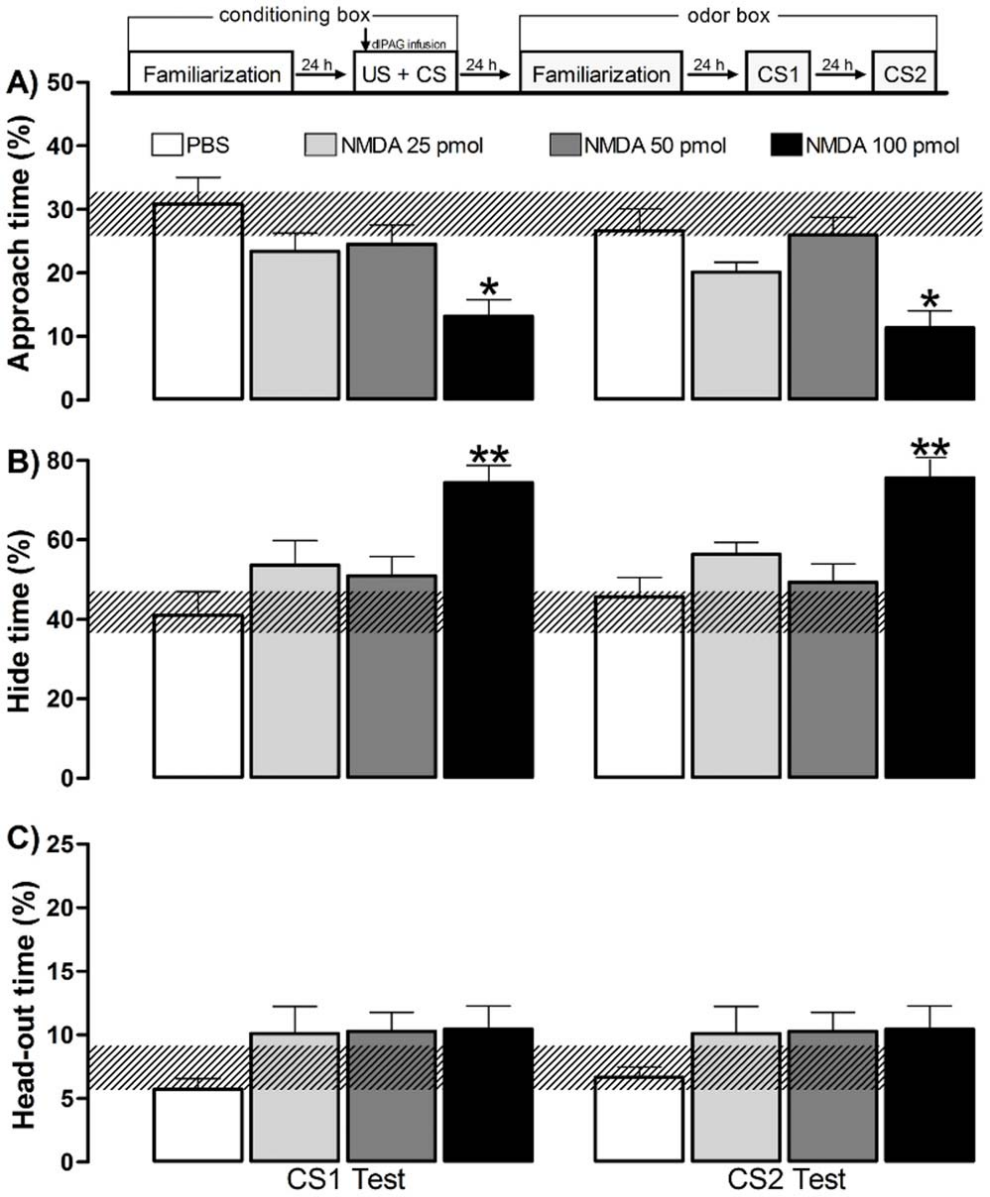
Experiment 1– Effects of the NMDA injection into the dlPAG in the acquisition of OFC. The experimental design used is outlined above the graph, where a vertical arrow shows the moment of the dlPAG infusion associated with amyl acetate odor during the conditioning session (10 min) in the conditioning box. Histograms represent the mean (+SEM) of the percentage of approach time (A), hide time (B), and head-out time (C) exhibited during OFC expression in an odor box. The hatched horizontal line represents the mean and the confidence limits (within 95%) for the familiarization session in the odor box. Subjects were grouped according to the different schedules of the dlPAG injection: PBS (n = 8), NMDA 25 pmol (n = 8), NMDA 50 pmol (n = 8) and NMDA 100 pmol (n = 8). CS1 represents the first-order CS exposure and CS2 represents the second-order context (no odor) exposure. *p<0.05 and **p<0.005 compared with the PBS control group (repeated measures ANOVA followed by Newman-Keuls test).

#### Experiment 3

This experiment was designed to evaluate the duration of AMYL odor exposure, in the conditioning session, necessary to support the acquisition of OFC. For this, the subjects were assigned to three groups: 1) PBS/odor group; 2) NMDA/odor/5 min; and 3) NMDA/odor/10 min. For the control group, rats received PBS injection and were paired during 10 min with AMYL odor (group 1, n = 8). For the other two groups, rats were microinjected into the dlPAG with NMDA and immediately exposed to the conditioning chamber with AMYL odor, for 5 min (group 2, n = 9) or 10 min (group 3, n = 8). Twenty four hours later, the expression of the defensive behavior was measured in three sessions (familiarization, CS1 and CS2, 24 h apart) in the odor box, as previously described.

#### Experiment 4

To outline the putative pathways from the dlPAG that would be able to support the OFC, we have examined the ascending projections of the dlPAG. Five animals received a single injection of *Phaseolus vulgaris*-leucoagglutinin (PHA-L, Vector Laboratories) into the dlPAG. First, they were anesthetized with a mixture of ketamine and xylazine (v/v; 1 ml/kg body weight), and then the iontophoretic injection of a 2.5% solution of PHA-L in 0.1 M sodium phosphate-buffered saline (pH 7.4) was made over a 10-min period through a stereotaxically positioned glass micropipette (10 µm tip diameter) by applying a +5 µA current, pulsed at 7-s intervals, with a constant-current source (Midgard Electronics). After a survival time of 14–16 days, animals were deeply anesthetized with sodium pentobarbital (40 mg/kg i.p.) and perfused transcardially with a solution of 4.0% paraformaldehyde in 0.1 M phosphate buffer at pH 7.4; the brains were removed and left overnight in a solution of 20% sucrose in 0.1 M phosphate buffer at 4°C. The brains were then frozen and five series of 40 µm-thick sections were cut with a sliding microtome in the frontal/transverse plane. One series of sections was processed for immunohistochemistry with an antiserum directed against PHA-L (Dako, Carpinteria, CA, USA) at a dilution of 1∶5000, and the antigen–antibody complex was localized by using a variation of the avidin–biotin complex system. In brief, sections were incubated for 90 min at room temperature in a solution of biotinylated goat anti-mouse IgG (Vector Laboratories, Burlingame, CA, USA; dilution 1∶200), and then placed in the mixed avidin–biotin horseradish peroxidase (HRP) complex solution (ABC Elite Kit; Vector Laboratories) for the same period of time. The peroxidase complex was visualized by a 10-min exposure to a chromogen solution containing 0.02% 3,3-diaminobenzidine tetrahydrochloride (DAB, Sigma, St Louis, MO, USA) in a 0.05 M Tris–buffer (pH 7.6), followed by incubation for 10 min in chromogen solution with hydrogen peroxide (1∶3,000), to produce a brown product. The reaction was stopped by extensive washing in potassium phosphate-buffered saline (KPBS; pH 7.4). The sections were mounted on gelatin-coated slides and then treated with osmium tetroxide to enhance the visibility of the reaction product. Slides were then dehydrated and coverslipped with DPX. An adjacent series was always stained with Thionin to serve as a reference for cytoarchitecture.

**Figure 4:**
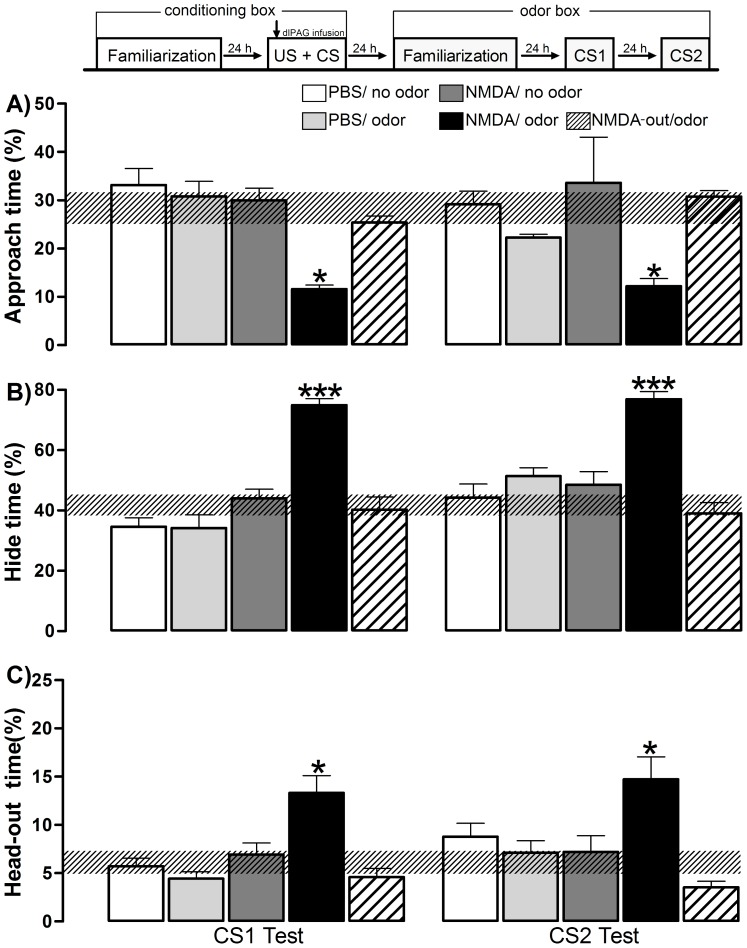
Experiment 2 - Selectivity of the dlPAG NMDA injection in supporting OFC. The experimental design used is outlined above the graph, where a vertical arrow shows the moment of the dlPAG infusion associated with amyl acetate odor during the conditioning session (10 min) in the conditioning box. Histograms represent the mean (+SEM) of the percentage of approach time (A), hide time (B), and head-out time (C). The hatched horizontal line represents the mean and the confidence limits (within 95%) for the familiarization session in the odor box. Subjects were grouped according to the different schedules of the dlPAG injection and training conditions during the acquisition phase: PBS/no odor (n = 8) - PBS infusion without amyl acetate odor pairing during conditioning; PBS/odor (n = 8) - PBS infusion with amyl acetate odor pairing during conditioning; NMDA/no odor (n = 8) - NMDA 100 pmol infusion without amyl acetate odor pairing during conditioning; NMDA/odor (n = 10) NMDA 100 pmol infusion with amyl acetate odor pairing during conditioning; and NMDA out/odor (n = 8) - NMDA 100 pmol infusion outside the dlPAG (in the midbrain reticular nucleus) with amyl acetate odor pairing during conditioning. CS1 represents the first-order CS exposure and CS2 represents the second-order context (no odor) exposure. *p<0.05 and ***p<0.0005 compared with the PBS/no odor group (repeated measures ANOVA followed by Newman-Keuls test).

#### Experiment 5

In this experiment, we tested whether the dlPAG ascending path to the medial hypothalamic defensive circuit, the main ascending target of the dlPAG, influences associative learning during OFC. Previous studies have shown that beta-adrenergic blockade of the dorsal premammillary nucleus (PMd; the main exit way for the hypothalamic defensive circuit to thalamo-cortical circuits involved in fear learning) prevents the contextual learning to cat odor [38]. Therefore, in Experiment 5, beta-adrenergic blockade of the PMd was performed immediately before the conditioning session when dlPAG-NMDA injection had been paired with AMYL odor. Rats microinjected into the dlPAG and the PMd received a second guide cannula (0.7 mm external diameter; 13 mm length) aimed at the PMd (coordinates from bregma: AP = 4.14 mm; ML = 0.8 mm; DV = 6.5 mm from the skull surface at an angle of 10°), according to *The rat brain in stereotaxic coordinates* [33]. (RS)-Atenolol (ATE; Tocris, Cookson, USA) was dissolved in 0.1 M in phosphate-buffered saline (PBS; pH 7.4), which alone served as vehicle control. The doses of ATE (40 nmol), the volume (0.3 µl) and rate (0.6 µl/min) of drug infusion were chosen based on previous studies [21], [38]. In all groups, PMd microinjections of PBS (n = 6) or ATE 40 nmol (n = 6) were performed, through a stainless steel needle (16.2 mm long with 0.35 mm external diameter) inserted into the guide cannula, five min before the animals received 100 pmol NMDA infusion in the dlPAG paired during 10 min with AMYL odor (CS). All groups were further analyzed in three consecutive days in the odor box, during a 10 min session each, according to the protocol previously described.

**Figure 5:**
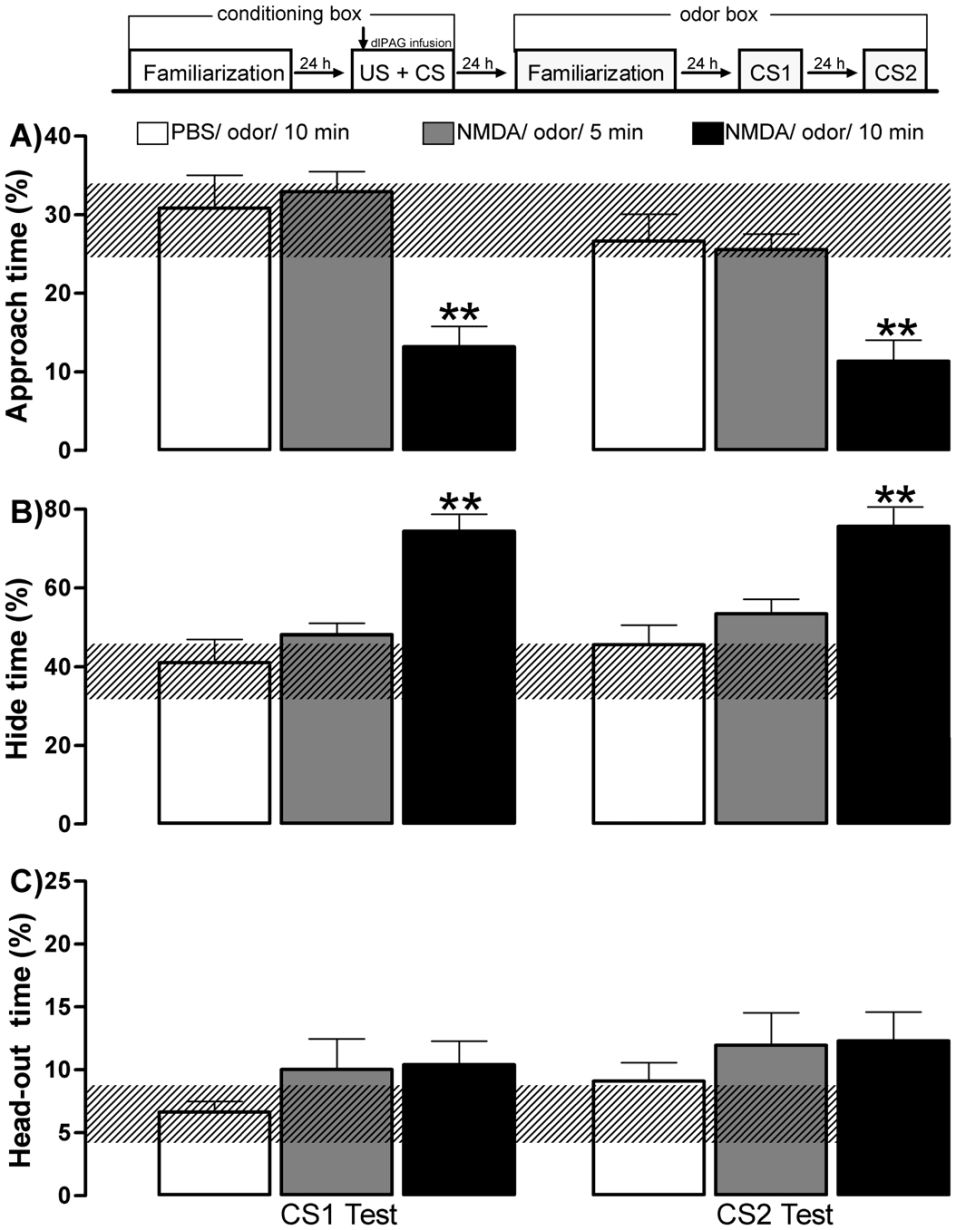
Experiment 3– Effects of the duration of odor exposure during dlPAG-NMDA OFC acquisition. The experimental design used is outlined above the graph, where a vertical arrow shows the moment of the dlPAG infusion associated with amyl acetate odor during the conditioning session (10 min) in the conditioning box. Histograms represent the mean (+SEM) of the percentage of approach time (A), hide time (B), and head-out time (C). The hatched horizontal line represents the mean and the confidence limits (within 95%) for the familiarization session in the odor box. All subjects were microinjected with NMDA 100 pmol and grouped according to the time interval of AMYL odor exposure during the conditioning session: 5 min (NMDA 5 min, n = 9) or 10 min (NMDA 10 min, n = 8). A group microinjected with PBS (n = 8) paired with amyl acetate odor during 10 min during the conditioning session was considered as control. CS1 represents the first-order CS exposure and CS2 represents the second-order context (no odor) exposure. *p<0.05 and **p<0.005 compared with the PBS 10-min group (control group; repeated measures ANOVA followed by Newman-Keuls test).

**Figure 6:**
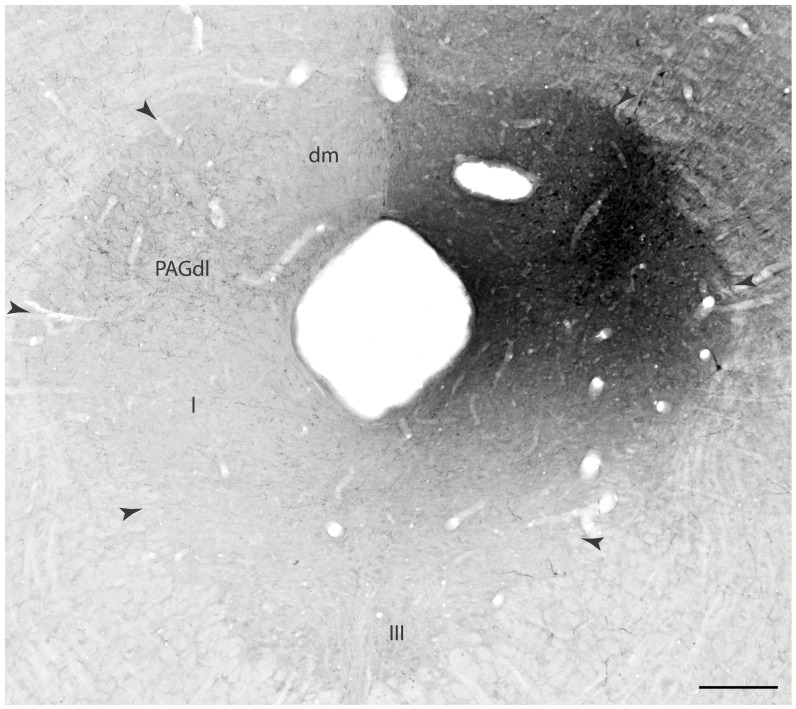
Experiment 4– Projections of the dlPAG. Bright-field photomicrograph, to illustrate the appearance of a PHA-L injection site for a representative PHA-L injection localized in the dlPAG (experiment dlPAG# 4). Note the plexus of PHA-L labeled fibers in the contralateral dlPAG. Abbreviations: III – oculomotor nucleus; PAGdl, dm, l – periaqueductal gray, dorsolateral, dorsomedial, and lateral parts. Scale bar = 250 µm.

### Histology to Verify Cannula Placement

At the end of the behavioral tests, subjects were deeply anaesthetized with sodium pentobarbital (40 mg/kg; Cristália, Brazil) and transcardially perfused with saline (0.9% NaCl) followed by a formaldehyde solution (10%) for 10 min. A volume of 0.2 µl of Evans blue dye (0.5%) was then applied through the same needle previously used in the experiments to mark the location of the drug microinjection. The brains were removed and post fixed overnight in10% formaldehyde solution and were transferred to a 30% sucrose solution for cryoprotection. Coronal sections (50 µm) were cut on a cryostat (Leica CM1850) and mounted on gelatin-coated slides. The sections were examined with an optical microscope to determine the injection sites delimited by the Evans blue dye.

Histological analysis confirmed that a total of 91 rats had accurate cannula placements in the dlPAG, while 12 rats had accurate cannula placements in both the dlPAG and the PMd. The dlPAG and PMd schematic injection site plotting, as well as representative photomicrographs showing the dlPAG and PMd cannula placements, are depicted on Figure 1. In rats receiving NMDA outside the dlPAG and paired with AMYL, the microinjections hit the midbrain reticular nucleus and were included in the dlPAG-out group (n = 8).

The figures were prepared for publication by using the Adobe Photoshop (version 4.0; Adobe Systems, Mountain View, CA, USA) for photomicrographs and the Adobe Illustrator (version 10.0; Adobe Systems) for line drawings. Only sharpness, contrast, and brightness were adjusted. Unless otherwise indicated, parcellation of the brain regions follows *Brain Maps* [39].

**Figure 7:**
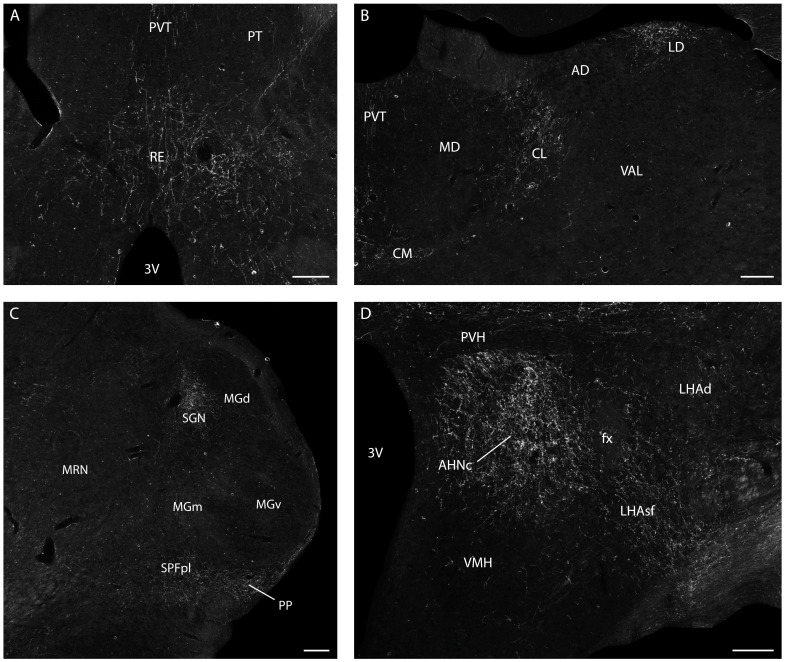
Experiment 4– Projections of the dlPAG. Dark-field photomicrographs showing the distribution pattern of PHA-L immunoreactive axons in the rostral nucleus reuniens (A), the intralaminar and lateral dorsal thalamic nuclei (B), the parvicellular subparafascicular, peripeduncular, suprageniculate and medial geniculate nuclei (C), and the anterior hypothalamic nucleus and subfornical region of the lateral hypothalamus (D). Abbreviations: 3 V – third ventricle; AD – anterodorsal nucleus thalamus; AHNc – anterior hypothalamic nucleus, central part; CL – central lateral nucleus thalamus; CM – central medial nucleus thalamus; fx – fornix; LD – lateral dorsal nucleus thalamus; LHAd – lateral hypothalamic area, dorsal region; LHAsf – lateral hypothalamic area, subfornical region; MD – mediodorsal nucleus thalamus; MGd, m, v – medial geniculate complex, dorsal, medial and ventral parts; MRN – midbrain reticular nucleus; PT – paratenial nucleus; PVH – paraventricular hypothalamic nucleus; PVT – paraventricular thalamic nucleus; RE – nucleus reuniens; SGN – suprageniculate nucleus; SPFpl – subparafascicular nucleus thalamus, parvicelular part, lateral division; VAL – ventral anterior-lateral complex thalamus; VMH – ventromedial hypothalamic nucleus. Scale bars = 200 µm.

### Statistical Analysis

Freezing behavioral data scored in the conditioning box following PBS or NMDA infusions were collapsed in two subsequent 5-min periods and analyzed by repeated measures analysis of variance (ANOVA), followed by the Newman-Keuls *post hoc* test.

Behavioral data (mean ± S.E.M.) scored in the odor box were also analyzed by repeated measures (CS1 and CS2) ANOVA, followed by the Newman-Keuls *post hoc* test. The approach time, the hide time and the head-out time during the 10-min session were scored min-by-min, and the 10-min collapsed data were transformed as the percentage of each measurement and used as dependent variables. The minimum level of statistical significance adopted was p<0.05. All statistical analyses were performed using the Statistica® software package (Version 10.1; StatSoft®, Tulsa, OK, USA).

**Figure 8:**
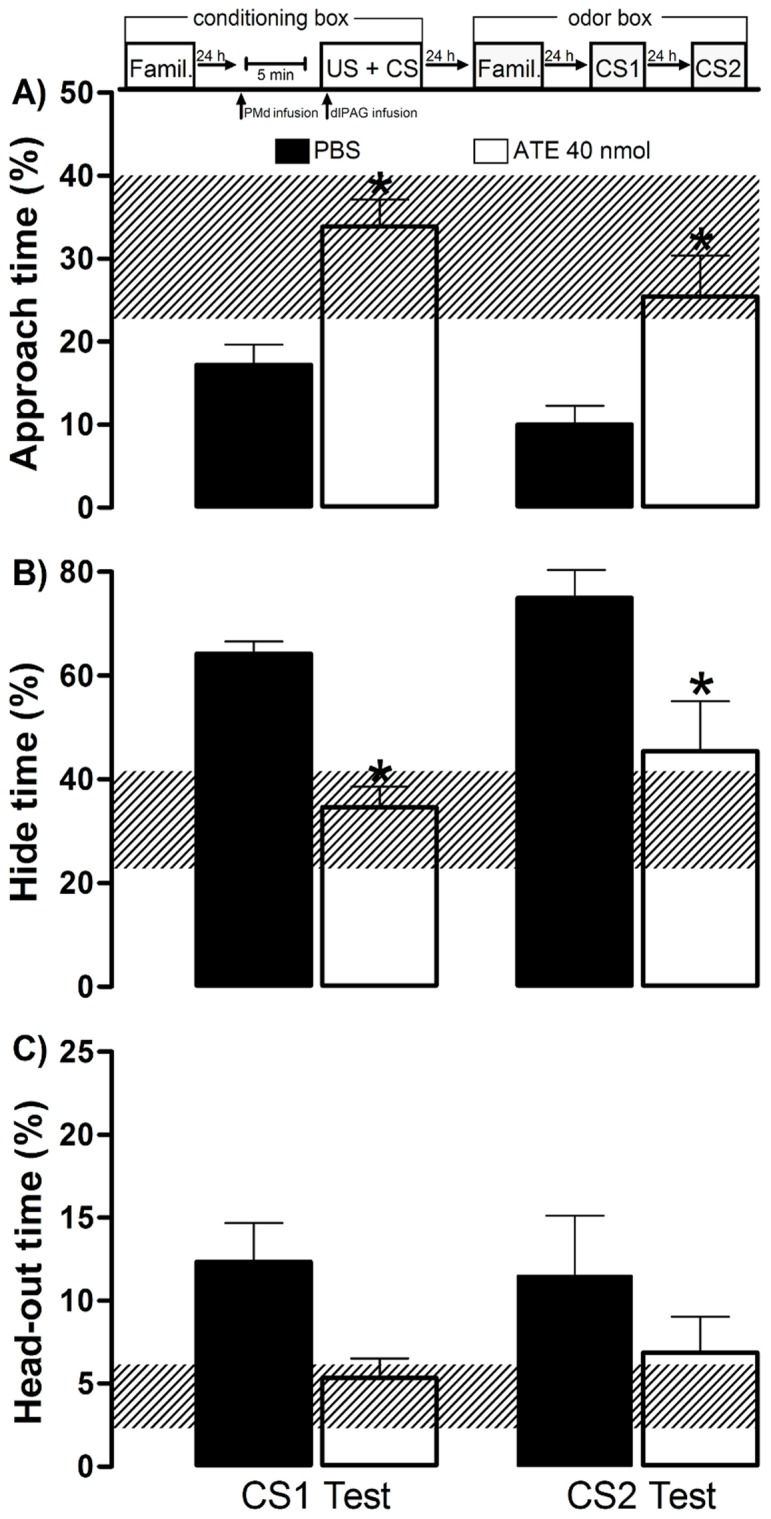
Experiment 5– Effects of dorsal premammillary nucleus blockade on dlPAG-NMDA OFC acquisition. The experimental design used is outlined above the graph, where vertical arrows show when the animals received the PMd and dlPAG infusions and were placed in a conditioning box with the olfactory CS. Histograms represent the mean (+SEM) of the percentage of approach time (A), hide time (B), and head-out time (C). The hatched horizontal line represents the mean and the confidence limits (within 95%) for the familiarization session in the odor box. Subjects were first submitted to an infusion into the PMd with PBS (PBS group, n = 6) or atenolol 40 pmol (ATE 40 pmol group, n = 6), and, after 5 min, were microinjected with NMDA 100 pmol into the dlPAG paired with amyl acetate odor during 10 min in the conditioning session. CS1 represents the first-order CS exposure and CS2 represents the second-order context (no odor) exposure. *p<0.05 compared with the PBS group (repeated measures ANOVA followed by Newman-Keuls test).

## Results

### Experiment 1– Effects of Crescent Doses of NMDA Injection into the dlPAG in the Acquisition of OFC

The defensive response exhibited during the 10 min conditioning session was represented by episodes of flight and jumping restricted to the first min after receiving NMDA into the dlPAG (Table 1), followed by increased freezing time maintained throughout the first 5 min period of observation. The % freezing time was scored and collapsed into two 5-min batches and are represented in Fig. 2. ANOVA detected significant treatment effect [F (3,28) = 3.99; p = 0.02], trial effect [F (1,28) = 8.86; p = 0.006] and a treatment versus trial interaction effect [F (3,28) = 3.56; p = 0.03] for the % freezing time. The *post hoc* test revealed a significant (*p*<0.05) increased % freezing during the first 5 min of the session for the subjects receiving NMDA 50 or 100 pmol into the dlPAG when compared to the PBS or NMDA 25 pmol groups. No statistical differences were detected among the groups when comparing the data acquired during the second 5-min period of the conditioning session. In addition, the *post hoc* test revealed a significant (*p*<0.05) decreased % freezing between the first and the second 5 min batch of the conditioning session for the groups receiving NMDA 50 or 100 pmol into the dlPAG. All NMDA doses used were able to elicit defensive responses, and subjects from the group that received the highest NMDA dose (100 pmol) consistently exhibited the greater magnitude for the three behavioral responses. As a whole, the defensive behavior intensity waned 5 min after the dlPAG-NMDA infusion for every dose used.

During the familiarization session in the odor box, the behavioral measurements showed no statistical differences among the groups. Therefore the hatched horizontal line in every figure represents the mean and the confidence limits (within 95%) for data obtained during the 10-min familiarization session in the odor box.

The OFC efficiency measured as the defensive behavior expressed during CS1 and CS2 is depicted in Figure 3. The ANOVA detected a significant treatment effect, represented by % approach time [F (3,28) = 7.92; p = 0.001] and % hide time [F(3,28) = 9.15; p = 0.001] but failed to detect differences for the % head-out time data. In the CS1 and CS2 sessions, the *post hoc* test revealed a significant decreased % approach time (*p*<0.05) and increased % hide time (*p*<0.005) for the subjects receiving NMDA 100 pmol into the dlPAG paired with AMYL odor, when compared to the PBS group. No statistical differences from the PBS control group were detected for the groups receiving NMDA 25 or 50 pmol. Furthermore, no statistical differences were detected when comparing the data acquired during the CS1 and CS2 sessions.

These results suggest that the 100 pmol of NMDA applied into the dlPAG worked as a useful US capable of supporting fear conditioning (US+CS association) and further context second order conditioning (CS1+CS2 association).

### Experiment 2– Selectivity of the dlPAG NMDA Injection in Supporting OFC

ANOVA performed with CS1 and CS2 data, depicted in Figure 4, detected a significant treatment effect for the % approach time [F(4,37) = 8.95; p = 0.0001], % hide time [F(4,37) = 28.47; p = 0.00001] and % head-out time [F(4,37) = 10.97; p = 0.00001]. A trial effect for the % hide time [F(1,37) = 19.08; p = 0.0001] and a treatment versus trial interaction effect for the % hide time [F(4,37) = 5.03; p = 0.002] were also detected by ANOVA. In the CS1 and CS2 sessions, the *post hoc* test revealed a significant decreased % approach time (*p*<0.05), and increased % hide time (*p*<0.0005) and % head-out time (*p*<0.05) for the subjects receiving NMDA 100 pmol into the dlPAG paired with AMYL odor when compared to the remaining four groups (PBS/no odor, PBS/odor, NMDA/no odor, NMDA-out/odor). Furthermore, no statistical differences were detected when comparing the data acquired during the CS1 and CS2 sessions.

Our results indicate that the dlPAG NMDA injection alone, not paired with AMYL odor, failed to induce generalized defensive responses during the behavioral testing session in the odor box. Moreover, our results pointed to the injection site specificity and showed that NMDA microinjection in the dlPAG, but not in adjacent regions, could be used as an effective US when paired with AMYL odor to promote OFC.

### Experiment 3– Effects of the Duration of Odor Exposure Necessary to the OFC Acquisition Induced by the dlPAG-NMDA Injection

In Experiment 3, shown in Figure 5, we tested the effects of different time intervals (i.e., 5 or 10 min) of AMYL odor exposure during the conditioning session for the acquisition of OFC.

**Figure 9:**
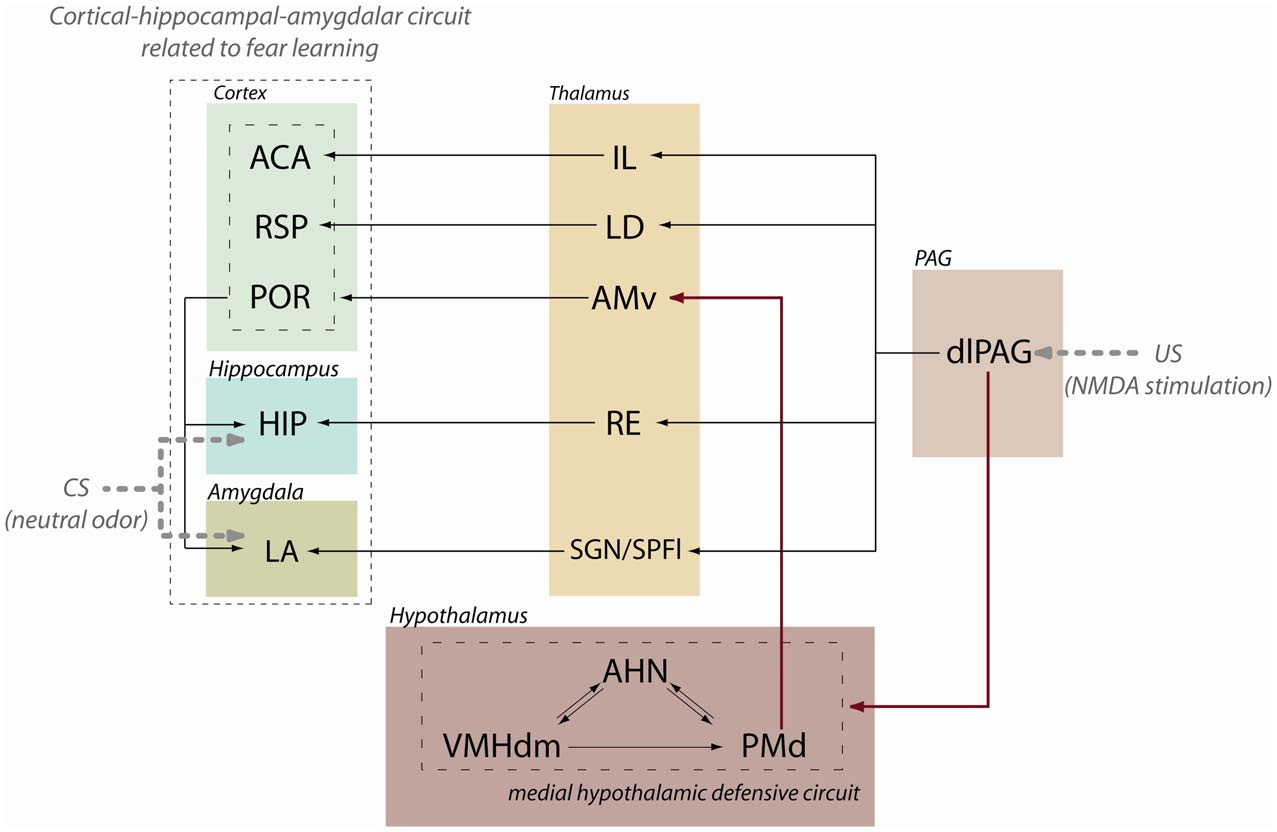
Summary diagram illustrating the dlPAG ascending projections to hypothalamic and thalamic targets influencing cortical-hippocampal-amygdalar circuits. Red lines indicate the dlPAG – medial hypothalamic defensive circuit – thalamic pathway, where we have shown that beta-adrenergic blockade of the dorsal premammillary nucleus impaired the acquisition of olfactory fear conditioning induced by the dlPAG-NMDA injection. Abbreviations: ACA – anterior cingulate area; AHN – anterior hypothalamic nucleus; AMv – anteromedial thalamic nucleus, ventral part; dlPAG – dorsolateral periaqueductal gray; HIP – hippocampal formation; IL – intralaminar thalamic nuclei; LA – lateral amygdalar nucleus; LD – lateral dorsal thalamic nucleus; PMd – dorsal premammillary nucleus; POR – postrhinal area;RE – nucleus reuniens; RSP – retrosplenial area; SGN – suprageniculate nucleus; SPFpl – subparafascicular nucleus thalamus, parvicelular part, lateral division; VMHdm – ventromedial hypothalamic nucleus, dorsomedial part.

ANOVA performed with CS1 and CS2 data detected a significant treatment effect in the % approach time [F (2,22) = 15.71; p = 0.00006] and % hide time [F(2,22) = 16.11; p = 0.00005]. No difference in % head-out time was observed. In the CS1 and CS2 sessions, the *post hoc* test revealed significant (*p*<0.005) decreased % approach time and increased % hide time only for subjects receiving NMDA 100 pmol into the dlPAG and paired with AMYL odor during 10 min, when compared to the PBS group. No statistical differences between the PBS group and subjects receiving NMDA 100 pmol into the dlPAG and paired with AMYL odor during 5 min were detected. Furthermore, no statistical differences were detected when comparing the data acquired during the CS1 and CS2 sessions.

These results suggest that, following the NMDA injection into the dlPAG, 10-min AMYL odor exposure seems an optimal time interval to support OFC acquisition.

### Experiment 4– Ascending Projections of the dlPAG

The results of the previous experiments support the idea that NMDA chemical stimulation of the dlPAG works as an effective US to support OFC. In experiment 4, we examined the dlPAG ascending projections to reveal potential targets involved in associative learning. In three experiments, the PHA-L injections labeled neurons mostly confined to the rostral dlPAG, at the level of the oculomotor nucleus, where we had previously aimed the NMDA injections (Fig. 6). In all of these experiments, a very similar pattern of anterogradely labeled fibers was observed, and of these, we chose PHA-L injection dlPAG# 4 as a prototype to illustrate our results, because the injection in that experiment labeled the most extensive population of cells in the dlPAG (Fig. 6). Ascending fibers from the dlPAG follow a ventral pathway through the midbrain reticular nucleus or continue rostrally through the periventricular system. From the injection site, a contingent of fibers enter the midbrain reticular nucleus and may continue rostrally to the caudal subthalamus and thalamus. Ascending fibers coursing through this pathway provide a dense projection to the peripeduncular nucleus and adjacent parvicellular part of the subparafascicular nucleus, as well as to the suprageniculate nucleus (Fig. 7C). In addition, a relatively small contingent of these fibers may project to the dorsal part of the medial geniculate nucleus (Fig. 7C).

The vast majority of ascending fibers from the dlPAG course through the periventricular system. At the transition between mesencephalon and diencephalon, dlPAG-ascending fibers provide a very dense terminal field in the periventricular region, including the precommissural nucleus. At diencephalic levels, fibers ascending through the periventricular system may be divided into two pathways, i.e., a dorsal path projecting to thalamic targets and a ventral one coursing through the subthalamic and hypothalamic regions. dlPAG-ascending fibers projecting to the thalamus provide a clear projection to the intralaminar nuclei, where the central lateral nucleus receives a substantial terminal field, and the other intralaminar nuclei contained only a relatively sparse number of labeled fibers (Fig. 7B). In addition, significant projections were also found to the rostral part of nucleus reuniens (Fig. 7A) and, to a lesser degree, to the rostrodorsomedial part of the lateral dorsal nucleus (Fig. 7B).

Ascending fibers coursing through the ventral path form a rather dense terminal field in the rostral part of the zona incerta. From the rostral zona incerta, fibers coursing through this path project to the hypothalamus and form a massive terminal field in the posterior and central parts of the anterior hypothalamic nucleus, and part of these fibers extend laterally, projecting substantially to the adjacent parts of the subfornical region of the lateral hypothalamic area (Fig. 7D). A relatively sparse number of fibers continue rostrally through the hypothalamus, projecting to the anterior part of the anterior hypothalamic nucleus and, to a lesser degree, to the medial preoptic area.

Finally, of particular relevance for the present study, we have also noted that the dlPAG provides important crossing projections to selected contralateral PAG sites, such as the contralateral dorsolateral column (see Fig. 6).

### Experiment 5– Beta-adrenergic Blockade of the Dorsal Premammillary Nucleus’ Effects on the Acquisition of OFC Induced by the dlPAG-NMDA Injection

As previously shown, the anterior hypothalamic nucleus, an integral element of the medial hypothalamic defensive circuit, represents the main ascending target of the dlPAG. In experiment 5, we tested whether the ascending input to the medial hypothalamic defensive circuit would interfere in the associative learning during OFC induced by the dlPAG-NMDA injection. To this end, immediately previous to the conditioning session (i.e.,when the dlPAG-NMDA injection was paired with AMYL odor), we performed a beta-adrenergic blockade of the dorsal premammillary nucleus, the main exit way for the hypothalamic defensive circuit to thalamo-cortical circuits involved in fear learning [15].

Data from this Experiment are shown in Figure 8. ANOVA detected a significant treatment effect for % approach time [F (1,10) = 17.1; p = 0.002] and % hide time [F(1,10) = 18.82; p = 0.001] during CS1 and CS2 sessions. No difference in % head-out time was observed. The *post hoc* test revealed a significant (*p*<0.05) decreased % hide time and increased % approach time for the subjects receiving ATE 40 nmol into the PMd when compared to the PBS injected group (Fig. 8). No statistical differences were detected when comparing the data acquired during the CS1 and CS2 sessions.

These results demonstrate that atenolol injected into the PMd is capable of impairing the acquisition of OFC promoted by the NMDA stimulation of the dlPAG.

## Discussion

In the present study, we have shown that NMDA stimulation of the dlPAG works as a useful US capable of supporting fear conditioning to a neutral olfactory cue. Next, combining anatomical and behavioral experiments, we were able to outline and test putative pathways mediating this effect.

The dlPAG is particularly mobilized during exposure to life threatening events, such as hypoxia [16], [17], cardiac pain [18] and predator threats [15]; and by chemically stimulating the dlPAG, we aimed at mimicking the neural activation in response to these events. Previous studies, using classical fear conditioning to sound- or light- conditioned stimuli, or conditioned place preference, have shown that either electrical or chemical stimulation of the dorsal PAG supports associative learning after several training sessions [19], [41], [42]. In the present study, we have been able to obtain a clear OFC using just one training session with a single dlPAG-NMDA injection, perhaps providing a closer match to what happens under a natural life threatening event. The present protocol was intended to provide a clearer understanding on how fear conditioning mechanisms work in response to life threatening events, and could represent a stepping stone in an attempt to uncover possible vulnerabilities or endophenotypes leading to defensive responses dysregulation in anxiety disorders, such as post-traumatic stress or panic disorders.

The present experimental protocol was based on the OFC task described by Kroon and Carobrez [29], in which the olfactory stimulus is isolated from the context by using two distinct chambers, i.e, the conditioning box for olfactory conditioning acquisition and the odor box for the OFC expression. We have initially tested the optimal NMDA dose and the appropriate duration of the training session to produce OFC. The best scores for the duration of the training session were found with a 10-min odor exposure, whereas shorter (5-min) AMYL exposure time proved ineffective in producing OFC. Immediately after receiving the NMDA injection, rats exhibited vigorous flight and jumping behavior restricted to the first min of observation followed by increased percentage freezing time, limited to the first 5-min session. The fact that NMDA infusion followed by a single 5-min AMYL exposure was not capable of supporting OFC suggests that, for the learning process, there is a need of an extra period of continuous AMYL exposure, when most of the overt defensive behavior has waned. It is reasonable to suggest that in order to provide the US-CS association following NMDA-dPAG injection, the odor exposure cannot rely exclusively on the overt defensive behavior presented during the first 5-min period, but also needs an extra 5-min period with a different defensive coping strategy. According to these facts, the continuous reduction of the freezing time during the second 5-min period seems to be a necessary step towards the OFC acquisition, when animals would be able to increase the environment exploration and enhance the discrimination of the olfactory CS. According to Steimer [43], a more active defensive strategy (as is expected during the first 5 min following the NMDA PAG stimulation) engages the subject in eliminating the source of threat to decrease the impact of stress and consequently the anxiety, whereas a less active defensive coping strategy (as seen during the last 5 min of odor exposure) is likely to increase the stress response and favor learning. Following NMDA infusion into the dlPAG, we were able to show a plethora of defensive responses during a 10-min period. Initially, as a result of NMDA receptor activation, a more active-like defensive behavior was observed, and the inability to form the US-CS association is indicative that contextual or cue learning seems unnecessary for this type of defensive coping style. However, as the NMDA-induced dlPAG activation declines, there is a behavioral shift towards a more conservative type of defensive coping style, increasing the subject appraisal of the olfactory CS, favoring the US-CS association. It seems reasonable to believe that the level of PAG activation may be critical to signal the appropriate coping style strategy and influence the resulting effects on fear learning.

The efficacy of the OFC was revealed in the odor box, by a series of risk/avoidance behavior that were also observed in experiments where rats had been confronted with cat odor [37], [38]. These subtle defensive behaviors displayed in the odor box included the time spent close to the CS, the time spent inside the enclosed compartment and the time spent heading out from the enclosed compartment, engaging in risk assessment behavior. In this protocol, the first odor box exposure, designated as familiarization session, has been carried out without AMYL odor, to test the behavior baseline and possible fear generalization in response to the new context. As previously discussed, during the conditioning session, we have obtained optimal parameters, which resulted in fearless behavioral baseline responses to a neutral odor, indicating no generalization, and providing clear fear conditioning responses when the olfactory cue (CS) was introduced in the first-order conditioning session (CS1). Lastly, in the CS2 test session, we were able to see that the CS1 was able to promote a contextual second-order conditioning (CS2), attesting to the robustness of the OFC. Notably, rats receiving NMDA outside the dlPAG during the conditioning session did not exhibit fear conditioning responses during either CS1 or CS2 sessions. It is important to mention that during the contextual exposition (CS2 session), the data values obtained were similar to those acquired during CS exposure, reinforcing the intrinsic biological significance of the dlPAG-NMDA activation. It is important to mention that following dlPAG-NMDA OFC no consistent alteration in %head-out time was detected during CS1 or CS2. These result contrast with the increased risk assessment behavior detected during CS1 and CS2 following the OFC obtained from the dorsal premammillary nucleus (PMd) activation (Pavesi et al., 2011). Based on the fact previously shown that the conditioning session, during dlPAG-NMDA activation resulted in much clear overt defensive behavior than during PMd activation, the unaltered risk assessment behavior could be due to the clear fearful strength represented by the dlPAG stimulation.

In agreement with previous tracing studies [40], we have found that the dlPAG provides particularly strong inputs to the anterior hypothalamic nucleus. The anterior hypothalamic nucleus, together with the dorsomedial part of the ventromedial nucleus and PMd, form a distinct circuit in the medial hypothalamus playing a pivotal role in integrating predator threats, and have been called the medial hypothalamic defensive circuit [15]. The present tracing findings also indicate that the influence of the dlPAG on the medial hypothalamic defensive circuit is further strengthened by strong projections to the rostral zona incerta and the subfornical region of the lateral hypothalamus, both of which known to provide important inputs to all elements of this medial hypothalamic circuit [44], [45]. Taken together, the connective data reveals that the medial hypothalamic circuit itself represents the major ascending target of the dlPAG. In line with this view, electrical stimulation aimed at the dlPAG induced a marked activation in the PMd [46], the main output way station of the medial hypothalamic defensive circuit, supporting the view that the dlPAG and the medial hypothalamic defensive circuit appear to operate in concert.

Notably, the medial hypothalamic defensive circuit has been shown to influence associative learning during contextual conditioning to predatory threat. Previous studies from our laboratory showed that pharmacological blockade of the PMd, with beta-adrenoceptor antagonist (atenolol), markedly influenced associative learning, linking cat odor to the related context [38]. Likewise, we have currently shown that the PMd beta-adrenergic blockade impaired OFC using chemical dlPAG stimulation as US likely to mimic a life threatening event. The PMd is thought to influence associative learning through its main thalamic target, i.e., the ventral part of the anteromedial thalamic nucleus (AMv) [15], [47]. Bilateral AMv lesions have been shown to block contextual, but not innate, fear responses to predator exposure [47]. The influence of the AMv on fear conditioning should involve its cortical targets, namely, the anterior cingulate and agranular retrosplenial areas [48], [49], both of which shown to support associative learning during fear conditioning [50], [51], [52], [53]. The role on associative learning of these cortical areas is likely to be mediated via the postrhinal area, through its strong inputs to the hippocampal formation and the lateral amygdalar nucleus [52], [53], [54], [55], [56].

As shown in Figure 9, apart from the ascending path to the medial hypothalamic defensive circuit, the dlPAG may potentially influence fear learning through a number of parallel thalamic paths. Our findings confirm previous anterograde tract tracing studies [57] and showed a projection from the dlPAG to the intralaminar nuclei, particularly aimed at the central lateral nucleus. The intralaminar nuclei, in turn, projects to the anterior cingulate area and form a path involved in fear learning [58]. According to the present findings, the thalamic targets of the dlPAG are much broader than previously reported (see [40]). We have seen that the dlPAG also projects to the nucleus reuniens, the lateral dorsal nucleus, the suprageniculate nucleus, and the parvicellular subparafascicular nucleus, all of which known to project to elements of the above described cortical-hippocampal-amygdalar path involved in fear conditioning, and, therefore, likely to serve the dlPAG to influence associative learning, as well (Fig. 9). The nucleus reuniens represents the main thalamic source of projection to the hippocampal formation [59]; the lateral dorsal nucleus provides a dense projection to the retrosplenial area [60], and the supragenicule and the parvicellular subparafascicular nuclei are important sources of inputs to the lateral amygdalar nucleus [61]. At this point, it remains to be investigated how each one of these dlPAG-thalamic paths may impact on fear conditioning.

There is an emerging view in the literature suggesting that the PAG plays a key role in influencing fear memory. Previous studies have shown that the ventrolateral PAG (vlPAG) seems critical to influence fear conditioning to painful stimuli through a path involving the intralaminar thalamic nuclei and the medial prefrontal cortex [62]. The present results expand this idea, and show that the dlPAG also supports fear conditioning, and should be particularly critical during fear learning to life threatening situations.

Altogether, the evidence suggests that the dlPAG is able to interfere with emotional judgments and mnemonic processes. This idea finds support in a meta-analysis of neuroimaging studies in humans that correlate affective behavior with the co-activation of the medial prefrontal cortex and a core limbic group, formed by the PAG, thalamus and hypothalamus, where the PAG activity seems critical to engage the thalamic and hypothalamic regions [63]. The remarkable similarities for the PAG activity across species, including humans, in emotional turmoil represents an important stepping stone for translational studies in fear and anxiety disorders.
